# Trajectories of HIV management among virally suppressed and unsuppressed female sex workers in the Dominican Republic: A comparative qualitative analysis

**DOI:** 10.1080/17482631.2023.2164947

**Published:** 2023-01-22

**Authors:** Virginia Savage, Hoisex Gomez, Martha Perez, Yeycy Donastorg, Deanna Kerrigan, Clare Barrington

**Affiliations:** a Department of Health Behavior, Gillings School of Global Public Health, University of North Carolina at Chapel Hill, Chapel Hill, North Carolina, USA; b HIV Vaccine Trials Research Unit, Instituto Dermatologico y Cirugia de la Piel, Santo Domingo, Dominican Republic; c Department of Prevention and Community Health, Milken Institute School of Public Health, George Washington University, District of Columbia, USA

**Keywords:** HIV, female sex workers, viral suppression, Dominican Republic, narrative analysis, stigma

## Abstract

**Purpose:**

Despite suboptimal HIV outcomes among female sex workers (FSW), limited research has been conducted on factors that impact viral suppression among this population. Examining narratives of HIV management, we examined how experiences of diagnosis, treatment initiation, and ongoing care behaviours shaped viral suppression outcomes over time.

**Methods:**

We conducted 20 in-depth interviews with FSW in Santo Domingo, Dominican Republic. Using narrative and thematic qualitative approaches, we developed analytic summaries and matrices to compare trajectories of managing HIV between suppressed and unsuppressed participants.

**Results:**

Regardless of suppression status, participants described similar narratives of overcoming initial challenges to HIV management through personal resilience and social support. Unsuppressed participants identified more delays in initiating antiretroviral therapy and more lapses in adherence due to less active acceptance of their HIV status and more persistent experiences of economic hardship and HIV stigma.

**Conclusions:**

We found that individual, interpersonal and structural factors, including stigma and economic precarity, differentiated trajectories towards viral suppression among FSW indicating the importance of multilevel interventions. Improved access to mental health services and social support could promote greater early acceptance of HIV status and progress towards viral suppression among FSW.

## Introduction

The final step of the continuum of HIV care and treatment, viral suppression improves quality of life and survival among people living with HIV. It also represents an important strategy in HIV prevention as virally suppressed individuals are less likely to transmit the virus (Kay et al., 2016; Skarbinski et al., 2015). However, reaching viral suppression is frequently a non-linear process, in which people living with HIV may transition in and out of care or experience periods of inconsistent adherence, which may prevent or lead to variability in viral suppression (Kay et al., 2016; Zulliger et al., 2018).

Accounting for eight percent of all new infections worldwide in 2019 (UNAIDS, 2020), female sex workers (FSW) living with HIV face numerous challenges to becoming virally suppressed. First, depression and other mental health issues are associated with delayed engagement with care following diagnosis among FSW (Payán et al., 2019; Zulliger et al., 2018). FSW who have experienced violence from intimate partners or clients are also less likely to be linked to HIV care and more likely to miss antiretroviral therapy (ART) doses and interrupt ART, among other treatment outcomes (Mendoza et al., 2017; Wilson et al., 2016). While not disclosing HIV status to partners and negative reactions to disclosure may affect ART initiation and retention in care (Schwartz et al., 2017; Zulliger et al., 2018), emotional and economic support and social cohesion can improve treatment outcomes (Kerrigan et al., 2017; Lancaster et al., 2016; Zulliger et al., 2015). Additionally, negative side effects of ART are associated with poor adherence (Glick et al., 2020; Zulliger et al., 2018), as are substance and alcohol use (Lancaster et al., 2016; Long et al., 2020; Muth et al., 2017; Zulliger et al., 2018). Structural factors such as stockouts of antiretroviral medications at health facilities, long waiting times at facilities, and sex work and HIV stigma, particularly from healthcare providers, are also barriers to optimal HIV outcomes (Lancaster et al., 2016; Wanyenze et al., 2017; Zulliger et al., 2015, 2018). Economic costs of transportation, testing and medical treatments, and adequate nutrition to consume with ART have also been identified as barriers to adherence and retention for FSW (Glick et al., 2020; Lancaster et al., 2016; Zulliger et al., 2018).

As a result of these multilevel factors, critical gaps remain in promoting viral suppression among FSW worldwide (Cowan et al., 2017; Lancaster et al., 2016; Mountain et al., 2014; Muth et al., 2017). In the Dominican Republic (DR), where FSW are disproportionately affected by HIV (CONAVIHSIDA, 2012), a 2019 study found while over 96.0% of FSW were on ART, only 76.2% had an undetectable viral load of less than 400 cells per cubic millilitres (Karver, Donastorg, et al., 2022) (CONAVIHSIDA, 2012. Possible explanations included that only 79.3% reported adhering to ART in the previous four days and 31.0% had missed an HIV care appointment in the previous six months (Karver, Donastorg, et al., 2022). Qualitative research has also found that FSW in the DR cycle in and out of care as they experience changes in financial security, ART shortages at clinics, changes in their physical health status, and variations in social support for accessing care (Zulliger et al., 2018). However, beyond describing barriers along the continuum of HIV care, minimal research in the DR and elsewhere has examined in-depth the processes that aid or obstruct FSW from becoming virally suppressed (Duff et al., 2016; Kerrigan et al., 2017; Long et al., 2020; Muth et al., 2017).

In this paper, we aim to gain a deeper understanding of factors affecting viral suppression by examining HIV narratives of virally suppressed and unsuppressed FSW in Santo Domingo, Dominican Republic. Using a combination of narrative and thematic analysis, we compared suppressed and unsuppressed FSW lived experiences of HIV diagnosis, treatment initiation, retention in care, adherence, and perceptions of factors contributing to their current suppression status. By examining how experiences with HIV lead to viral suppression, we aim to inform strategies to promote optimal HIV outcomes and overall wellbeing among female sex workers in the Dominican Republic and other settings.

## Methods

### Parent study

**Figure 1.**
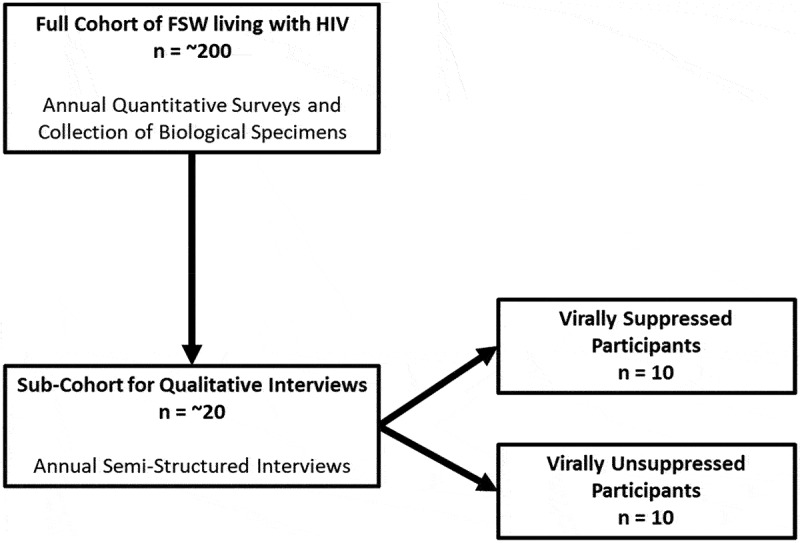
Overview of parent study sampling design.

We analysed baseline qualitative data from a longitudinal, mixed methods study on stigma, social cohesion, and HIV outcomes among FSW in Santo Domingo (R01MH110158). For quantitative aims of the parent study, socio-behavioural surveys and biological specimens were collected every 12 months with a cohort of 201 FSW to assess the impacts of various social determinants on HIV treatment adherence and viral suppression outcomes. At these same time intervals, qualitative semi-structured interviews were conducted with a subset of 20 participants to contextualize quantitative findings and gain further insight into lived experiences of managing HIV over time (Figure 1).

### Sample and recruitment

Participants were recruited by peer healthcare navigators in person or via phone. Navigators also used referrals from clinics, key informants, and participation in the research team’s previous interventions. Eligibility criteria included being at least 18 years of age, reporting having exchanged sex for money within the previous month, and being HIV positive as confirmed by a rapid test. For the sub-cohort of FSW participating in qualitative semi-structured interviews, participants were purposively sampled from the full cohort on being virally suppressed (defined as 400 copies per cubic millilitre) (*n* = 10) or unsuppressed (anything higher than 400 copies per cubic millilitre) (*n* = 10) (Figure 1). We determined that we reached data saturation with 20 participants, which was also a feasible sample size for the longitudinal component of the study.

### Data collection

All interviews were conducted in a private room at the office of the Instituto Dermatológico y Cirugía de Piel, a research and healthcare facility in Santo Domingo. Study procedures were approved by the Institutional Review Boards at Johns Hopkins University in the US and the Instituto Dermatológico y Cirugía de Piel. All participants provided informed consent prior to engaging in interviews, surveys, and the collection of biological data. Participants also received a contextually appropriate financial incentive for their participation.

### Data analysis

Our analysis includes data from the first round of qualitative interviews from the parent study. These interviews were conducted in October and November of 2018 by the second and third authors (HG and MP). Both interviewers had conducted research with some participants in past studies, which facilitated establishing trust and rapport. Open-ended questions were designed to elicit participants’ narratives of their diagnoses and experiences living with HIV, while probing particularly on the role of social determinants, including social cohesion and HIV and sex work stigma, in affecting their health and wellbeing. All interviews were audio-recorded and transcribed verbatim in Spanish by Dominican transcribers with previous qualitative research experience.

Analysis was conducted in Spanish by the first and sixth authors (VS and CB), both American academics fluent in Spanish and with professional experience in the DR. In accordance with Sandelowski’s recommendations for early analysis (Sandelowski, 2000), we began by “getting a sense of the whole,” reading transcripts and listening to audio recordings of interviews several times to develop a general impression of the themes, storylines, and voices salient in each interview. We then created an analytic summary for each participant that described their HIV diagnosis, care and treatment, social networks, and experiences of sex work. These summaries are living documents that have since served to integrate and expand upon narrative and themes at later rounds of interviews (Barrington et al., 2021).

We inductively identified key topics in analytic summaries to develop a codebook to examine common determinants of health and progression through the HIV care continuum. We conducted multiple rounds of coding using the qualitative coding software ATLAS.ti (v.8) first using the initial codebook and later with interpretive and in vivo codes. To refine understanding of HIV trajectories, we created a matrix of key themes from coding across different chapters of participants’ HIV narratives. Finally, we compared cases of participants who were suppressed at the time of the baseline interview with those who were not. This iterative approach allowed us to compare experiences and themes across interviews while keeping those themes situated within the participant’s broader narrative of living with HIV (Barrington et al., 2021). We use case examples of one suppressed and one unsuppressed participant throughout each section of the results to reflect their trajectories and present findings in a holistic manner, while also integrating examples and quotes from other participants. We also present a table describing key themes that we observed across interviews (Table 2).

## Results

Mean participant age was 38.9 years (range 21 to 53 years) and time living with HIV ranged from three to 38 years (Table 1). Nearly half of the participants believed that they contracted HIV from a former husband or steady partner. There was great variation in the time that the ten virally suppressed participants had maintained undetectable viral loads, with four women only becoming suppressed in the previous year and the other six having maintained viral suppression for longer periods of time, in one case up to 17 years. Of the 10 participants who were not virally suppressed, eight had been living with HIV for over ten years. Four unsuppressed participants believed that they had been virally suppressed in the past but had not maintained that suppression status.

**Table I.** Participant information.

Viral Suppression (<400 copies per cubic millilitre)	#	%
Yes	10	50
No	10	50
Ages	#	%
21-28	3	15
29-36	3	15
37-44	10	50
45-53	4	20
Years living with HIV	#	%
<5	3	15
6-9	4	20
10-14	6	30
15-20	4	20
>21	2	10
unclear	1	5
Acquired HIV from:	#	%
Spouse or steady partner	9	45
Sex work client	2	10
Mother-to-Child Transmission	1	5
Participant is not sure	4	20
Participant did not describe	4	20
Number of living children and financially dependent grandchildren	#	%
1	4	20
2	8	40
3	7	35
4	1	5

Overall, participants largely inhabited economically vulnerable situations. Several participants earned income from sources outside of sex work, such as small businesses, healthcare, and cleaning. Forms of sex work also varied among participants: while some women worked out of establishments or met clients on the street, others coordinated with clients through online messaging services or phone, and others worked with regular clients exclusively. Participants’ involvement in sex work also evolved throughout their narrative of living with HIV. While seven women described working at sex work establishments or the street in the past, they had since reduced their involvement in sex work in recent years, meeting clients less frequently or working exclusively with a small number of regular clients. Two participants stated that they had not done sex work in the previous few months, due to fear of violence, fear of exposure to illness, or stigma related to their HIV status or occupation as a sex worker.

Finally, every participant had children, many of whom were still financially dependent. The number of living children ranged from one to three, and four participants also financially supported or provided care for their grandchildren. One participant had great-grandchildren, and another was pregnant at the time of the interview. Four participants had lost a child since being diagnosed with HIV.

Regardless of suppression status, participants shared narratives of adaptation in which they overcame mental and physical health challenges to improve their outlook on life and HIV management. In the following sections, we present the case studies of one virally suppressed and one unsuppressed participant that embody many of the common themes from these adaptation narratives. Both participants were given pseudonyms in these case studies to further protect confidentiality. Drawing from the case studies and from other participants’ narratives, we explore similarities and differences in the challenges that virally suppressed and unsuppressed participants faced in accepting their HIV diagnosis and managing their condition in the past. We then explore how most participants were able to overcome those challenges through processes of self-reflection and gaining social support. Finally, we compare factors that suppressed and unsuppressed participants believe to be impacting their current suppression status. A summary of key themes throughout participants’ narratives can be found in Table 2.

**Table II.** Key themes in participant narratives.

Theme	Description	Illustrative Quote
Mental health challenges	Participants’ experiences of depression, grief, and emotional distress concerning their HIV status. These challenges often centred around fears of death, concerns for their children’s future, and guilt over transmission to others.	*I lost all of those pounds because I was not eating, I had a relationship with a person and I was thinking, I was saying and that this person had his wife, and so I blamed myself, I did not know, I didn’t know anything about anything and I say “my God!” in thinking about this other person, I was thinking about my children, when I would see my children, I would say that they are going to be left alone and such [continues sobbing] and maybe this is why I feel so good*. (unsuppressed)
Denial of diagnosis	Participants’ rejection of their HIV diagnosis, as well as their more general unwillingness to accept their condition.	*Before I was not really taking responsibility, I wasn’t taking it in, I wasn’t understanding what was happening to me, eh I used to take the medication but it wasn’t having an effect on me, I would go to talks at places, and I would listen, or rather I would hear what they were saying but I wouldn’t give any importance to the advice, I didn’t give any importance to what they were saying to me, because I was thinking that I didn’t have anything*. (virally suppressed)
ART side effects	Fears and actual experiences with ART side effects and the impact of both on treatment initiation and adherence.	*I cried a lot because thinking that … I thought that I was going to die, because the people were saying that it was not very advisable to take those medications, that sometimes instead of making you feel better they would put you in a worse condition*. (virally suppressed)
Conflicts and tension	Lack of support, arguments, and rejection from family members, friends, or neighbours that affected participants’ HIV management.	*Participant: Because in June was when she [her daughter] left and I kicked [her ex-partner] out more or less in May, I left him in May more or less around then […]* *Interviewer: So before that you were fine and from there that was when you got worse?* *Participant: Yes, because from there that was where I started to think more about it […] to neglect caring for myself. I didn’t want to comb my hair, because I, I recognized it, one day I recognized: “I am falling into a depression.” I didn’t want to comb my hair, I didn’t want to put on makeup, I didn’t want to*. (virally suppressed)
Loss of loved ones	Participants’ experiences of caretaking for ill family members or grieving the passing of partners or family members, which affected their mental health and HIV treatment adherence.	Interviewer: *And why did you stop the medications?* Participant: *The neglect and also the worry for [her husband who passed away], for taking care of him, I was not going to my house, I was taking everything there including even what I would be eating, as he was almost not eating, I would divide the same food for him and we would both eat, the few things that he was eating, because he was just tasting food, he was not eating anything … ”*(unsuppressed)
Social support	Emotional, financial, and logistical support from friends, family members, healthcare providers, or support group facilitators that aided participants’ commitment and ability to manage HIV.	*I left until one day they found me and called me at where I was working and they tell me that I was needing to go, that if I was gambling with my health […] and etcetera, until I went, then I renewed treatment, I started again from zero, and from there I started going appointment after appointment, appointment after appointment, until I met [a counsellor for the support group], she brought me here. Here I have shared a lot*. (unsuppressed)
Internal reflection processes	Psychological processes to accept their HIV status and improve management, including reflections on their desire to be present for their children, understanding that HIV is a manageable condition, and empowerment to take initiative in managing their health.	*Everyone that used to see me, I don’t know, they would say that it was tuberculosis too, ay but she is going to die, she is going to die, and one day I stood up and I said, “who is going to die, me?” And I stood up from my bed […] and here I am 100% moving forward*. (virally suppressed)
Acceptance of HIV status	Participants’ expressions of accepting and making peace with their HIV status after initially denying or lamenting their diagnosis.	… *and about my health condition, the truth is that I feel well because I have tried to overcome, and I have tried to accept myself, you know that when one accepts themselves, things change because one can fight illnesses with the mind, when one does not accept it, you already have to go and what else is there to say (laughs) …* (virally suppressed)
Pro-active self-care	Participants’ efforts to prioritize their health and wellbeing including healthy nutrition, sleep habits, and avoidance of drug use, among other behaviours.	Interviewer: *Have you ever stopped taking the medications?* Participant: *“Never, it can happen to me that I can miss a few hours because I go out, sometimes I think that I have it in my pockets or I have it in the wallet and I don’t have it, but immediately when I arrive, I take it, and I haven’t missed picking up my medications either, I do not mess around with that.”* (virally suppressed)
Prioritizing mental health to improve ART adherence	Participants highlighted the importance of maintaining a positive attitude and resisting HIV stigma to maintain optimal ART adherence.	*that I have strengthened myself mentally, or rather that I am not allowing anything to affect me, that nothing and nobody affects me* (virally suppressed)
Improved adherence	Participants’ improvements in following their ART regimen and attending regular medical appointments since their HIV diagnosis.	*No, nothing, now I have to take them for all my life because now for me to have HIV is better than to have cancer […] because cancer kills a person more quickly and HIV at least has medication and I drink my medication as prescribed, it is not going to kill me, I can die from something else, but from HIV no*. (unsuppressed)
Not thinking about HIV	Participants’ efforts to live a “normal life” as if they did not have HIV.	*Well, my tranquillity that after I come to the program, I have been more stable, calm I don’t think much about that which, or I don’t give thought to the virus in my body*. (unsuppressed)
Economic instability	Psychological and logistical barriers to managing HIV that participants experienced due to their economic situation. Examples included food insecurity, cost of transportation or medications, housing instability, and demands of sex work that compromised adherence and health.	*“Fast” means that when I don’t have money, I become like a “desperate person,” […] I would go out to look for money and I would forget, and the pills would stay in the house*. (unsuppressed)
HIV stigma	Participants’ fears of being rejected by family and friends due to their HIV status, as well as their lived experiences of discrimination. This theme also captured internalized stigma, or participants’ feelings of shame and low self-esteem because of their HIV status.	Interviewer*: What would happen that you would get that way that you did not want to take the pills? […]* Participant: *Sometimes one thinks about why they have [HIV], sometimes one gets focused on “ay why do I have that, I am not a good person,” or many people when they think that a person has it they ignore the person, sometimes it gets into the person’s head. One time I wanted to take my own life*. (unsuppressed).

### Initial challenges with adapting to life with HIV

#### Case study #1: Maribel, unsuppressed

Maribel was diagnosed with HIV along with her husband with whom she had two young children. As they both had HIV, she believed they could maintain “*a normal life*,” and she was linked with care through a friend at MODEMU, a local sex worker rights organization. However, her husband suffered depression and passed away within three months of his diagnosis. Maribel explained that “*my life collapsed after he passed away*.” She began to feel apathetic and engage in destructive behaviours:

*I went through a time when I was saying that whether I was dead or alive was all the same to me, and I told myself, I let myself go, in the sense that I wasn’t taking care of myself, ehh … I wasn’t eating … eee, I was waking up often in the street, drinking, 24/7 because I was saying that that disease is not going to kill me, I am going to be killed from fumes on the street or getting hit by a drunk driver*

She was also fearful to begin ART as she knew someone whose HIV status was exposed after an allergic reaction. As a result, she delayed starting ART and her health deteriorated rapidly. Once she eventually began treatment, Maribel experienced negative side effects that compromised her adherence. Fear of stigma also obstructed her from accepting her HIV status and affected her motivation for practicing adherence and self-care.

#### Case study #2: Esther, virally suppressed

Esther contracted HIV from a partner with whom she was living. In addition to suffering power imbalances in the relationship from their 20+ year age difference, she believes that her partner withheld knowledge of his HIV status from her. After losing weight and becoming ill, Esther got tested for HIV against her partner’s wishes. She received a positive result, ended their relationship, and despite experiencing distress and fear, sought care shortly after her diagnosis to improve her poor state of health.

Her first prescription of ART caused severe allergic reactions that required hospital care. Her health continued to deteriorate until she became bedridden at her father’s house for several months. Rather than offering support, her family members began preparing for her funeral, even bringing supplies to the house. This treatment fuelled her beliefs that she would die imminently, and she became apathetic to self-care and adherence.

After recovering from this experience, Esther later became afraid of passing the disease to others, and she isolated herself at her mother’s house in the country. During this time, her ART adherence was irregular: “*I would come from … the country to pick up my medications here, but I would not take them at the correct time because I would say: ‘but what for?’*” This period of very poor adherence lasted for around five years and her health worsened.

The cases of Maribel and Esther highlight the various challenges that participants faced following diagnosis as they initiated HIV care. Like Maribel and Esther, most (18/20) participants, regardless of their suppression status, shared similar narratives of emotional distress struggling to accept their condition after their diagnosis. Perceiving HIV as a death sentence, they described experiencing depression, denial, and fear for their children’s futures (Table 2). These reactions were often compounded by simultaneous experiences of personal loss, fears of side effects from taking ART, and lack of social support (Table 2). As a result, like Maribel and Esther, several participants delayed initiating treatment and suffered lapses in ART adherence in their initial years of living with HIV.

Delays in initiating treatment were more common among participants who were not virally suppressed at the time of the interview (*n* = 6) than among suppressed participants (*n* = 2). For example, an unsuppressed participant nearly rejected life-saving treatment for a complication of childbirth after learning her diagnosis. She described:

*When they arrived there I looked at the paper at once, and at once I saw that it said “positive.” Immediately I got worse and I took out the blood they were going to give me, I didn’t want saline, I didn’t want anything, to the point in which I signed a paper telling them to not give me anything, and I called some people to take my daughter who was 14 years old and my son. I said that his dad, that his family on his father’s side would take him. I thought I was going to die, I didn’t want to hear anything from anyone, I didn’t want them to give me medications or anything.*

Though another hospital patient eventually encouraged her to accept treatment for the complication, her perspective about the severity of her HIV diagnosis did not improve, and fear of death and stigma inhibited her from seeking treatment for two years. Echoing Maribel’s narrative, another unsuppressed participant “*felt that I was going to go crazy*” and considered taking her life from despair over her diagnosis. This participant also equated her HIV diagnosis to a death sentence and struggled with questions about her children’s future. She delayed starting treatment for several months, despite experiencing severe declines in her health. Conversely, for suppressed participants, post-diagnosis fear and distress had a reduced impact on treatment initiation. As demonstrated in Esther’s case, many suppressed participants began treatment quickly to alleviate severe illnesses or to prevent mother to child transmission of the virus.

Even after they initiated treatment, both unsuppressed and suppressed participants continued struggling to accept their HIV status and adapt to their new condition. In their initial years of receiving ART, both suppressed (*n* = 5) and unsuppressed (*n* = 7) participants experienced lapses in adherence for several reasons. First, family rejection, lack of social support, and partner or family conflicts occurred at times in which they were already struggling to accept their diagnosis and learn to manage their condition (Table 2). Like Esther, some reacted by isolating themselves for fear of stigma or passing HIV to someone else. Consequently, lack of support and isolation aggravated poor mental health states, diminishing participants’ motivation to protect their health and practice optimal adherence. Second, various participants suffered negative side effects of ART including severe reactions that required medical attention, as Esther experienced, as well as milder effects, as Maribel described, which discouraged their adherence (Table 2). Some participants mentioned forgoing medications which caused drowsiness or interacted with alcohol or drugs as they negatively affected their sex work income.

A third factor that contributed to lapses in adherence was caretaking responsibilities in their households. Caretaking-related stress led participants to forget their medication and limited time and resources to acquire medication (Table 2). As demonstrated in Maribel’s narrative, grief from the loss of loved ones also fuelled depression and apathy towards adherence and self-care (Table 2). Another unsuppressed participant experienced extreme stress from caring for her son who also had HIV as well as from financial instability. Unable to pay her rent and suffering HIV stigma in her neighbourhood, she was forced to move without support from family or friends. Her son also passed away shortly after the move. She described: *“Because I was thinking about it a lot, when the boy died, ‘ay I am going to die like the boy, so skinny’.”* Not only did her son’s passing cause her grief, but it also cemented her fear of death as imminent and inevitable, which increased her apathy towards adherence. Additionally, she experienced adverse side effects from ART and negative interactions with her doctor who emphasized that she would die and threatened to send her to another facility if her adherence did not improve. As a result of these intersecting challenges, she did not take her medications and her overall health declined for several months. Other unsuppressed participants like Maribel also reflected on how similar experiences of anticipated and experienced stigma negatively affected mental health, which lead to not picking of medications or seeking social support that would help adherence (Table 2).

This participant’s experience also highlights how economic hardship and the corresponding stress created challenges related to HIV care and overall wellbeing (Table 2). Another unsuppressed participant reflected on how economic stress required her to become more involved in sex work following her diagnosis, which negatively affected her health. Economic hardship also limited participants’ access to food to take with ART to reduce side effects. While economic challenges were mentioned across participants, their negative impacts on HIV care and treatment were more notable among unsuppressed participants.

Overall, for both virally suppressed and unsuppressed participants, initial challenges with care resulted from multiple intersecting factors. Negative mental health, loss of loved ones, lack of social support, conflict with family members, and negative side effect of ART hindered Maribel, Esther, and many other participants from adapting to living with HIV and progressing through the care continuum. However, while nearly all participants suffered emotional distress following their HIV diagnosis, unsuppressed participants like Maribel more frequently experienced delays in initiating care and lapses in ART adherence, whereas Esther and other suppressed participants began treatment more rapidly. The negative impact of economic hardship and stigma was also more pronounced in these initial care challenges among unsuppressed participants.

### Reversing the narrative: overcoming challenges to improve outlook and HIV management

#### Case study #1: Maribel, unsuppressed

Eventually, Maribel overcame delays and lapses in care with social support and personal resolve. First, a friend helped her to seek care and learn to live with her condition. She also began seeing a psychologist who alleviated her concerns about ART. In learning to accept her condition, Maribel also underwent a period of personal reflection:

*After that, I myself realized that everything is mental and if you get in your head that you are going to die from something even though you don’t have to die from that thing. And after that I sat down to reflect, that I had an aunt who died from HIV and in reality, she lasted about thirty years or so with the virus. When I realized that she had that virus, afterwards I started talking with my grandmother and my uncles about her, and they told me, no girl, she lasted so many years with that disease and nobody knew … so I told them, but my aunt was living in that condition, and why can’t I live like my aunt lived?*

Discussing the example of her aunt’s experience with her family, Maribel gained confidence that she could manage her condition and keep her HIV status concealed if she chose. Additionally, she secured sources of income outside of sex work, which increased her self-esteem. These increased feelings of agency helped her to adopt a more positive mentality and commitment to practicing optimal adherence.

#### Case #2: Esther, virally suppressed

While bedridden from the reaction to ART, Esther had a dream, in which she realized that she had the power to improve her health and she gained motivation to be present for her children. To her family’s surprise, she stood up from the bed and began eating. During this time, Esther also received support from a doctor who visited her at home and treated the ongoing ART side effects. She described:

*“When I said to him in the afternoon, “I want more food,” my son, the one that died, “mami is eating!’ and that’s how it happened. From there I started to take my medications as prescribed, from there I left for my house where I could take care of children, when I could be present for them.’*

Moving into her own house raised her self-esteem and helped distance her from conflicts with other family members. Esther also began seeking the support of a counsellor who helped her learn to cope with stigma and accept her condition more.

Later, during the five-year period of poor adherence, she eventually began to learn more about HIV and how condoms could prevent transmission. This knowledge empowered her to “*start her life*,” to overcome her self-imposed isolation and begin sex work again. Earning her own income and negative changes in her health also motivated her to commit more fully to taking her ART as prescribed.

Both Maribel and Esther shared narratives of overcoming challenges to HIV management that centred around personal empowerment, social support, and healthcare provider interactions. Echoing both case studies, several other participants underwent personal empowerment processes through which they changed their perspective of HIV and gained motivation to better manage their condition (Table 2). These processes often entailed reflections on their identities as mothers, as seen in Esther’s narrative. Whereas despair at the thought of orphaning their children had initially affected care, several women improved their adherence realizing that “*it would be worse*” for their children if they died. For example, after suffering years of denial of her condition, depression, and family conflict that compromised her adherence, one suppressed participant had also become bedridden. She recalled a moment of clarity:

*And I said: I have to get up from this bed, or rather I said that for me, to myself, I have to stand up from here because I have two children and I have to fight for them, if I will not do it, nobody will do it, because maybe a grandmother can take care of them, or an aunt can help them one day but no one is going to do it like me, because I am their mother…*

Realizing her role in her children’s lives, she gained motivation to improve her adherence. Other participants, like Maribel and Esther, amended their fatalistic views of HIV by educating themselves more. Learning more about HIV from health care providers, workshops, and support groups among other sources, participants began not only to overcome apathy towards self-care and adherence, but also to realize their own agency in managing their health.

Second, both suppressed and unsuppressed participants highlighted that emotional, informational, or instrumental support from friends, family, or support groups enabled them to overcome challenges to ART initiation and adherence (Table 2). For example, like Maribel, several unsuppressed participants overcame delays initiating ART when family members or friends provided encouragement, financial support, and accompaniment to health facilities. Another unsuppressed participant sought care with informational support and encouragement from her neighbour, whose husband had died from HIV complications. Other participants received encouragement, cohesion, and advice from support groups for sex workers living with HIV. For example, one unsuppressed participant described:

*“When I started the [care] processes and getting involved and talking and looking at other cases, and this and that, and the talks there at MODEMU and such, that was when I started motivating myself and thinking that if I were to die it would be worse because who would I leave my children with? So … ”.*

Like other participants, finding cohesion and support from other women living with HIV aided her throughout her processes of reflection and empowerment.

In addition to family and friends, health care providers also helped participants overcome challenges to HIV care (Table 2). In some cases, psychologists aided in reducing internalized and anticipated stigma, as described by Maribel and another unsuppressed participant:

*… if people were laughing, I would say it was about me, around then the people could say whatever thing, I would say it was about me, if you were there and were giving me a kiss and tomorrow you were ignoring me I would say that it was about me and because of that I would start to cry. But after I came here [the counsellor] helped me a lot, she made me realize about living for myself, that it is a disease, now it doesn’t matter to me that they say I have this, now it doesn’t seem like much.*

Like for Esther, other health care providers went out of their way to help several participants overcome barriers to care and adherence. For example, despite her deteriorating health and despair, one suppressed participant struggled to begin treatment as her initial care providers kept citing the need for further tests. Eventually, another doctor stepped in to prescribe ART and educate her about adherence, actions which the participant believes kickstarted rapid improvements in her health and mental outlook. Esther and another participant described improvements in mental health and adherence due to home visits or personalized education from a health care provider. With these sources of support, participants began to accept their condition, learn more about living with HIV, and focus on adherence and other health behaviours to work towards achieving viral suppression.

For most participants regardless of suppression status, improvements in HIV management were driven by a convergence of these individual and interpersonal factors. As highlighted in Maribel and Esther’s narratives, intrapersonal processes of reflection and empowerment often occurred alongside or as the result of receiving social support or assistance from medical personnel. Mental health also centred prominently in participants’ stories of improving care behaviours, with positive mental health framed as a determinant of optimal adherence.

### Living with HIV: managing adherence and wellbeing

#### Case study #1: Maribel, unsuppressed

At the time of the interview, Maribel perceived herself as “*more stable*” both mentally and with her HIV management. She believed that a positive attitude enabled treatment adherence and self-care, and she safeguarded her mental health by actively avoiding thoughts about her HIV status. However, her viral load has remained detectable. Despite improvements in her mental state and adherence, she continued to suffer stress and shame from experiences of stigma. Crying during the interview, she lamented:

*“[crying] I feel bad because it is not the same, there are people that see you as fine. There are people that don’t. There are people that judge you, there are people that don’t. Sometimes they judge you without knowing.” […] “Because they say, or at least to me they have said, that I am this way because I was looking for it, and it was not that I was looking for it, I was not looking for it, it came to me unforeseen, God wanted it to be that way, so I feel bad because people judge without knowing.”*

She believed this stress prevented her from achieving viral suppression and optimal health. Additionally, financial instability caused Maribel to forgo eating for multiple days to prioritize providing food for her children. During those times, she experienced severe dizziness from the medications that kept her from getting out of bed. Though discouraged by her doctor, she coped by temporarily discontinuing her medication.

#### Case study #2: Esther, virally suppressed

Whereas the demands of sex work, financial stress, stigma, and prioritizing her children had initially compromised her adherence and viral load for several years, Esther credited her viral suppression to her improved mental health, increased economic stability, and prioritization of self-care. To cope with experiences of stigma or family conflict that caused her stress and affected her adherence, she purposefully ignored “gossip” and sought out social support from her psychologist and peers.

*Because it’s that with gossip one forgets even their medications, forgets to feed themselves, forgets even to bathe themselves, forgets even to brush their hair, because they are in problems, in gossip, in backwardness, and I learned all of this, [her psychologist] explained all of this, to let it go, that you have this, I let it go, let it go, every time that they were calling me whatever thing, I turned my back, after I saw that it was crushing*

When asked if there were any factors that could affect her health status, Esther responded: “*I believe no because I do not stop at what they will say. I know that first, first me, second me, third me, and fourth me. If I am keeping up my medications and my nutrition everything is ok*.” She focused primarily on her own agency as the determinant of her viral suppression.

In describing their current health, both Maribel and Esther highlighted improvements in their HIV management and overall wellbeing since diagnosis. Such positive perspectives of current health were echoed by nearly all participants regardless of suppression status. Having overcome negative emotions after their diagnoses, most participants felt that they had achieved greater acceptance of their HIV status which in turn helped improve their adherence and overall self-care (Table 2). However, in reflecting on their approaches to sustaining adherence and suppression, Esther and other suppressed participants described more agency and pro-active attitudes to overcoming challenges than did Maribel and most unsuppressed participants. For example, most suppressed participants credited their undetectable viral loads to their dedication to mental health care (Table 2). Like Esther, many described that they actively rejected negative thinking, judgement, or other barriers to self-care that might affect viral suppression (Table 2). One suppressed participant even specified “*mental hygiene*” as a key HIV management practice, which she defined as “*like when they say something ugly to you, you try to get rid of it so that it doesn’t get to you, you try to dodge it*.” Overall, mental health care practices among suppressed participants reflected a higher level of acceptance of their HIV status and helped them cope with stigma and other challenges.

Similarly, suppressed participants like Esther frequently emphasized that self-care helped them to sustain suppression (Table 2). Along with adherence, this self-care entailed maintaining healthy nutrition, good home hygiene, and healthy sleep habits. Relating to sex work, self-care included forgoing drugs and alcohol, avoiding late nights, and insisting on condom use with clients. Despite experiences of stigma and logistical barriers, some suppressed participants expressed confidence in their ability to overcome challenges to self-care by “*fighting*” for their health. This motivation to “fight” often stemmed from continued reflection on their roles as mothers. For example, whereas one suppressed participant once experienced sadness for her children’s quality of life, she explained her current motivation to prioritize self-care as:

*I return to this and repeat it to you, it was in in thinking, the desire to live, the desire to keep my children moving forward, the desire to fight for what I have and what God gave me, the opportunity to be a mother, or rather … I fight for what I want and what God gave me which are my children and for that I am still here*

Driven by the love for her children whom she called “*the motor of her life*,” she resolved to prioritize her health and adherence despite stigma and barriers to care. As a result, she had maintained an undetectable viral load for approximately six years prior to the interview.

Conversely, although Maribel and other unsuppressed participants also identified their improved outlooks and mental health as key determinants of adherence, they frequently described more passive approaches to accepting their condition and managing their health. Rather than emphasizing their own agency in HIV care, unsuppressed participants like Maribel frequently described efforts to avoid thinking about their HIV status (Table 2). For example, one unsuppressed participant explained that she maintained her mental health and adherence by not thinking about it, “*I do not think much about it, or I don’t give much thought to that there is a virus in my body*.” In general, negative emotions and “*thinking*” in general were portrayed as directly harmful to one’s health and HIV management. One participant even discussed lethal outcomes of thinking saying: “*when one feels, one grieves, starts to think, think, think- this kills*.” For these participants, living a “*normal life*” entailed giving minimal attention to their HIV status except for treatment adherence and directly related behaviours like nutrition.

As demonstrated in the case studies above, another key comparison between participants was the impact of ongoing economic hardship, which featured more prominently in the narratives of unsuppressed participants (Table 2). Whereas Esther directly attributed her viral suppression to her increased economic stability, Maribel described ongoing financial insecurity which compromised her adherence at the time of the interview. Such economic hardship limited attending appointments and ART adherence, and was connected to poor mental health and limiting feelings of agency, as demonstrated in the case of one unsuppressed participant:

*… what is complicating things for me right now, I owe, I owe a lot, when they are coming to charge me to threaten me and I feel bad because of this, this is what is complicating my life a bit, but apart from this I feel good […] Ay yes, there are moments when I feel without luck, I cannot stand up from the bed but I ask God and he gives me strength and I stand.*

Additionally, for other participants like Maribel, food insecurity compromised adherence due to the side effects of taking ART on an empty stomach. Financial vulnerability also drove participants’ continued involvement in sex work and irregular eating patterns, both of which affected mental health, medication adherence, and viral suppression. Several unsuppressed participants detailed specific demands of sex work that also affected their adherence such as irregular work hours, pressure from clients to engage in drug or alcohol use, and difficulty in taking medications in the presence of clients.

Finally, more unsuppressed participants identified discrimination and stigma as ongoing barriers to optimal HIV management (Table 2). One unsuppressed participant referenced that stigma and fear of judgement prevented her from attending medical appointments and picking up medications. For other participants, stigma contributed to the financial vulnerability that affected their adherence. For example, one unsuppressed participant had been fired from a café due to her HIV status, and the resulting economic stress was detracting from her mental health at the time of the interview. Without fixed employment, she also continued to earn her income from sex work, where irregular work hours affected her adherence. Other unsuppressed participants like Maribel continued to feel judged and treated unfairly for their HIV status, feelings which fostered psychological distress and limited motivation to manage their health.

## Discussion

Regardless of viral suppression status, most participants described overcoming periods of depression, stress, or other mental health challenges to accept their condition and adopt a positive outlook on their HIV diagnosis. These trajectories were similar among virally suppressed and unsuppressed participants, and most women credited improvements in HIV management and general health at least in part to these improvements in mental health and attitude towards their condition. For all participants, self-reflection and social support from health personnel, friends, and family also fostered positive changes in mental health and medication adherence. Despite these similarities, unsuppressed participants more frequently experienced lapses in adherence and delays in treatment initiation. Furthermore, unsuppressed participants highlighted more experiences of economic hardship and HIV stigma that persistently detracted from wellbeing and adherence.

A key finding from this analysis was the impact of mental health on participants’ wellbeing, HIV care, and viral suppression. Regardless of suppression status, nearly all participants experienced mental health challenges following their HIV diagnosis, which led many to delay treatment initiation or practice poor adherence. Numerous studies have documented that a positive HIV diagnosis may trigger psychological distress and depression among FSW and other populations (Bhadra et al., 2020; Payán et al., 2019; Perrett & Biley, 2013; Zulliger et al., 2018) and that depression may be associated with suboptimal ART adherence and lower CD4 counts (Weinstein & Li, 2016). In the DR, two qualitative studies among women living with HIV (WLH) discovered high levels of psychological distress and depression that were most severe after diagnosis, as we observed in our analysis (Payán et al., 2019; Rael, Carballo-Diéguez, et al., 2017). Similarly, evidence from the DR (Kerrigan et al., 2021; Zulliger et al., 2018) has highlighted that depression and other mental health challenges may compromise linkage to HIV care and ART adherence among FSW and WLH more generally. For participants in our analysis, mental health challenges typically detracted from HIV care by fuelling apathy towards their health condition, a phenomenon which has been documented in other populations in the United States (Kamat et al., 2016, Babicz et al., 2021). Conversely, participants’ improvements in HIV management paralleled processes of overcoming mental health challenges and adopting more positive outlooks on their condition. These findings align with previous qualitative studies in the DR and elsewhere that have identified social support, reflection on motherhood or survival identities, and increased health literacy as catalysts for “turning points” for WLH to improve mental health and acceptance of their diagnosis (Medley et al., 2009; Payán et al., 2019; Rael, Carballo-Diéguez, et al., 2017).

Another key result from our analysis was the impact of social determinants, in particular stigma and poverty, on HIV care, especially among unsuppressed participants. Stigma and financial difficulties limited participants’ access to information about care, created fear to attend health appointments or carry medications in public, limited resources to pay for transportation or care, and caused deprivation of food to take with ART (Glick et al., 2020; Lancaster et al., 2016; Zulliger et al., 2018). In addition to these direct effects, stress from stigma and economic hardship, as well as from social conflict or personal losses, often exacerbated or coincided with participants’ mental health challenges to reduce individual agency and impact care. As has been found among people living with HIV in other settings (Gibson et al., 2011; Weinstein & Li, 2016), our findings indicated that stress may be an important determinant of HIV treatments outcomes among FSW in the DR. By acting as a mechanism through which structural issues impact individual care behaviours, stress may also help explain quantitative associations that have been found in the DR between internalized stigma and income with depression and anxiety among FSW (Kerrigan et al., 2021; Rael, Davis, et al., 2017). Overall, our results underscore the need to address issues of economic inequality and HIV stigma that underlie the social vulnerability persistently obstructing some participants from becoming suppressed.

Our results also indicate that mindfulness may impact viral suppression not only by influencing participants’ mental health, but also in buffering the effects of structural barriers on HIV care behaviours. Though the term “mindfulness” was not mentioned explicitly, we observed that suppressed and unsuppressed participants expressed different levels of acceptance of their HIV status. Whereas unsuppressed participants often avoided dwelling on their condition to prevent depression and apathy towards adherence, virally suppressed participants described more of an “attitude of acceptance or non-judgement towards experience” of HIV, a key component of mindfulness as has been described in other studies (Feldman et al., 2007).

Past researchers have made contrasting arguments about how “not thinking about” living with a chronic condition, such as HIV, might impact health behaviours and outcomes. First, another study in the DR found that adults living with type-2 diabetes used “not thinking about it” as an adaptive coping strategy for managing their condition and protecting their mental health (Sadeghzadeh et al., 2021). By keeping themselves occupied with work and social interactions, focusing on their diet and medical recommendations, and turning to religion, participants in that study were able to avoid rumination on their condition and stress that might affect their diabetes management (Sadeghzadeh et al., 2021). However, our findings align more closely with various studies among people living with HIV that have characterized avoidance or distancing oneself from the condition as a maladaptive coping mechanism that negatively impacts mental health (McIntosh & Rosselli, 2012). For example, similar to our observations, another qualitative study in the DR argued that the focus on maintaining “a normal life” might prevent people living with HIV from facing and gaining control of the complex factors related to HIV management (Barrington et al., 2018). With more mindful acceptance of their HIV status, suppressed participants in our study were able to adopt strategies of pro-actively rejecting stigma and prioritizing mental health and wellbeing, which have been noted as benefits of mindfulness-based interventions (Kerrigan et al., 2018, 2021). Our results also align with and expand on quantitative analyses from the parent study that found associations between higher levels of mindfulness, lower levels of depression and anxiety, lower levels of internalized stigma, and higher likelihood of viral suppression (Kerrigan et al., 2021). It is also possible that higher levels of mindfulness may have increased suppressed participants’ resilience to socioeconomic stressors (Van der Gucht et al., 2015), which could explain why those issues were mentioned less frequently in their interviews. Overall, having been shown to improve mental health outcomes, quality of life, and ART adherence in various settings (Kerrigan et al., 2018; Scott-Sheldon et al., 2019; Tumminia et al., 2020), interventions to increase mindfulness may be effective to address stress reduction and increased acceptance of HIV status among FSW in the DR (Kerrigan et al., 2021; Scott-Sheldon et al., 2019).

In addition to mindfulness, social support from the health care system and participants’ social networks often enabled both suppressed and unsuppressed participants to overcome structural and psychological challenges to optimal care. In line with previous research in the DR and elsewhere (Lancaster et al., 2016; Zulliger et al., 2015, 2018), health system supports in the form of positive interactions, counselling, and education from health care providers increased participants’ agency in HIV management and helped participants overcome logistical barriers to care. These findings also expand on quantitative associations previously found between patient-provider communication and increased odds of viral suppression and ART adherence (Karver, Donastorg, et al., 2022). Additionally, in line with previous research on peer healthcare navigators and social support interventions among FSW living with HIV in the DR (Karver, Barrington, et al., 2022; Kerrigan et al., 2020), peer social support and cohesion in our analysis helped participants to overcome stigma and structural barriers to HIV management while increasing their agency in accessing care and advocating for their health. These findings support confidence in the ability of peer navigator programs and other social support initiatives to improve the health of FSW living with HIV. Though previous research has documented the positive impacts of intimate partner support on HIV management (Lancaster et al., 2016), more research may also be needed on strategies to increase support and involvement of FSW family members, considering their key roles in participants trajectories.

This analysis has limitations. First, while we knew the viral suppression of status of each participant based on viral load testing, some participants expressed limited comprehension or familiarity with the term “viral suppression,” which could foster unreliability in narratives of achieving and losing viral suppression in the past. Second, the scope of experiences described in our analysis is limited by the small number of participants in the cohort. Similarly, as most of the participants lived in Santo Domingo and were involved in other intervention and research activities, it is possible that their shared experiences differ from those of sex workers living in more rural areas or who have minimal contact with health care system.

Despite limitations, this analysis has implications for future initiatives to support FSW living with HIV in the Dominican Republic and beyond. We found that delays in treatment initiation, lapses in adherence, and challenges with achieving viral suppression often resulted from a convergence of multilevel issues, rather than from a single determinant. This convergence of factors speaks to the need for comprehensive, multilevel interventions not only to increase self-acceptance, agency in managing HIV, and social support for FSW, but also to reduce structural inequalities that jeopardize FSW health and viral suppression outcomes. Such interventions should particularly emphasize the promotion of accessible mental health care services to help FSW accept their HIV status and cope with stress from stigma, financial hardship, and other sources. By improving individual HIV viral suppression, multilevel interventions might also function as prevention measures to help control HIV transmission. Results also speak to the importance of examining the continuum of HIV care as a non-linear process, in which different factors may intersect and affect participants throughout multiple continuum stages of being diagnosed, beginning treatment, and maintaining adherence to sustain viral suppression. Further research among populations with more diverse experiences is needed to identify determinants of HIV management throughout those different stages. Quantitative studies could also aid in determining statistical interactions and cumulative effects of those determinants on viral suppression and care outcomes among larger sample sizes.
